# A novel integrated gene coexpression analysis approach reveals a prognostic three-transcription-factor signature for glioma molecular subtypes

**DOI:** 10.1186/s12918-016-0315-y

**Published:** 2016-08-26

**Authors:** Sujuan Wu, Junyi Li, Mushui Cao, Jing Yang, Yi-Xue Li, Yuan-Yuan Li

**Affiliations:** 1 School of Biotechnology, East China University of Science and Technology, Shanghai, 200237 China; 2 Shanghai Center for Bioinformation Technology, 1278 Keyuan Road, Shanghai, 201203 China; 3 Key Laboratory of Systems Biology, Institute of Biochemistry and Cell Biology, Shanghai Institutes for Biological Sciences, Chinese Academy of Sciences, Shanghai, 200031 China; 4 Shanghai Industrial Technology Institute, 1278 Keyuan Road, Shanghai, 201203 China; 5 School of Life Science and Technology, Tongji University, Shanghai, 200092 China; 6 Shanghai Engineering Research Center of Pharmaceutical Translation, 1278 Keyuan Road, Shanghai, 201203 China

**Keywords:** Glioma molecular classification, Prognostic biomarker, Differential coexpression analysis, Differential regulation analysis, Glioma carcinogenesis

## Abstract

**Background:**

Glioma is the most common brain tumor and it has very high mortality rate due to its infiltration and heterogeneity. Precise classification of glioma subtype is essential for proper therapeutic treatment and better clinical prognosis. However, the molecular mechanism of glioma is far from clear and the classical classification methods based on traditional morphologic and histopathologic knowledge are subjective and inconsistent. Recently, classification methods based on molecular characteristics are developed with rapid progress of high throughput technology.

**Methods:**

In the present study, we designed a novel integrated gene coexpression analysis approach, which involves differential coexpression and differential regulation analysis (DCEA and DRA), to investigate glioma prognostic biomarkers and molecular subtypes based on six glioma transcriptome data sets.

**Results:**

We revealed a novel three-transcription-factor signature including AHR, NFIL3 and ZNF423 for glioma molecular subtypes. This three-TF signature clusters glioma patients into three major subtypes (ZG, NG and IG subtypes) which are significantly different in patient survival as well as transcriptomic patterns. Notably, ZG subtype is featured with higher expression of ZNF423 and has better prognosis with younger age at diagnosis. NG subtype is associated with higher expression of NFIL3 and AHR, and has worse prognosis with elder age at diagnosis. According to our inferred differential networking information and previously reported signalling knowledge, we suggested testable hypotheses on the roles of AHR and NFIL3 in glioma carcinogenesis.

**Conclusions:**

With so far the least biomarkers, our approach not only provides a novel glioma prognostic molecular classification scheme, but also helps to explore its dysregulation mechanisms. Our work is extendable to prognosis-related classification and signature identification in other cancer researches.

**Electronic supplementary material:**

The online version of this article (doi:10.1186/s12918-016-0315-y) contains supplementary material, which is available to authorized users.

## Background

Glioma, a broad category of brain and spinal cord tumors, has very poor prognosis because of its infiltration and heterogeneity. Precise classification of glioma is essentially required during each patient’s therapeutic treatment. There are three major types of glioma traditionally classified by affected glial cells: astrocytomas, oligodendroglioma and ependymoma. From classification criterion given by World Health Organization (WHO) in 2007, glioma is then categorized according to its grade which is determined by pathologic evaluation [1]. This classification method of glioma based on morphologic and histopathologic knowledge is associated with clinical outcomes and used to anticipate the prognosis of patients. However, the traditional classification scheme is subjective, and is highly relying on individual experiences. Moreover, patients within same histopathologic subtype and grade may show different clinical outcomes [2]. Therefore, further investigation of glioma molecular characteristic factors is necessary for clinical diagnosis and targeted treatments.

Recently, more classification methods based on molecular variation are developed with the rapid progress of high-throughput technologies. These molecular classification methods enhance the profiling of cancer subtypes and promote precision medicine. A large amount of biomarkers including mRNA expression patterns [3–6], DNA methylation markers [7], microRNA expression signatures [8], copy-number profiling patterns [7, 9] and proteome profiling patterns [10, 11] have been found to identify glioma molecular subtypes. For instance, The Cancer Genome Atlas (TCGA) identified clinically relevant subtypes of glioblastoma according to an 840-gene signature [3]. Wei Yan et al. studied whole genome gene expression data from samples of the Chinese Glioma Cooperative Group and provided a prognostic classification scheme featured with 1577 genes [4]. A nine-gene signature in glioma patients is defined by Bao et al. based on mRNA expression data analysis [5]. In Sun et al.’s work, a glioma classification scheme based on coexpression modules centered by EGFR and PDGFRA was suggested [6].

In order to narrow down the searching space, some of these methods started from known glioma related genes. Still taking Sun et al.’s work as an example, they investigated the co-expressed genes of EGFR and PDGFRA and defined them as the molecular biomarkers of glioma subtypes [6]. However, this strategy retains little chance of finding out novel factors. Moreover, differential expression analysis (DEA) is used in most classification schemes, which proves to be capable of discovering biomarkers successfully [12–15]. In recent years, differential co-expression analysis (DCEA) and differential regulation analysis (DRA) are emerging in the transcriptome analysis domain as a prospective complement to traditional differential expression analysis (DEA) [16]. By looking at changes in gene expression correlation, DCEA and DRA offer hints about the disrupted regulatory relationships or abnormal regulations specific to the phenotype of interest [17–19]. In contrast, traditional DEA calculates expression level changes of individual genes between phenotypes, and has less chance to discover causal regulatory factors. Following this sense, we developed a glioma classification scheme which integrated DCEA and DRA to nonnegative matrix factorization (NMF) [20] clustering method. This integrated approach is supposed to have stronger potential to unveil prognostic signatures than that traditional differential expression analysis has. By this approach we can discover biomarkers which are more relevant to regulation mechanisms underlying glioma carcinogenesis.

In this study, we analysed 6 public transcriptome glioma data sets and identified three glioma prognosis-related transcription factors, AHR, NFIL3 and ZNF423. The expression values of these featured genes divided patients into three distinct molecular subtypes which were characterized by significantly different clinical outcomes and gene expression patterns. We investigated the relevance of the three-TF signature to regulation mechanisms of glioma carcinogenesis and suggested AHR, NFIL3 and ZNF423 as promising biomarkers for not only glioma molecular subtype diagnosis but also clinical treatment. Our novel integrative gene coexpression analysis approach is also extendable to prognosis-related molecular classification and signature identification in other cancer researches.

## Methods

### Data sets

Six glioma transcriptome data sets including GSE4290, GSE16011, GSE4412, Tiantan data set, Rembrandt data set and TCGA RNA-seq data set, were used in this study (Table 1). GSE4290, GSE16011 and GSE4412 data sets were gathered from Gene Expression Omnibus (GEO) [21–23]. TCGA RNA-seq data set was obtained from the TCGA data portal and the mRNA expression data of Glioblastoma multiforme (GBM) and Brain Lower Grade Glioma (LGG) were merged. We downloaded raw data (CEL files) of the Rembrandt data set [24] and merged all data sets by using R package ‘affy’ [25] with normalization index of mas5.0. The Tiantan data set was downloaded from Chinese Glioma Genome Atlas [4]. If expression value of a certain gene was missing in a particular data set, we ignored this gene in the follow-up analysis.

**Table 1:** Six glioma data sets used in the study

Data sets	Platform	Component of samples	Use
GSE4290	GPL570	157 glioma (AII 7, AIII 19, GBM 77, OII 38, OIII 12, unknown 4), 23 epilepsy	Used for DCEA and DRA analysis
GSE16011	GPL8542	284 glioma (PA 8, AII 13, AIII 16, GBM 159, OII 8, OIII 44, OAII 3, OAIII 25),8 normal adult brain samples	Training set for searching for DRA-based signature
Rembrandt	GPL570	521 gliomas (A 148, GBM 228, O 67, OA 11, unknown 67), 21 epilepsy	Training set for searching for DRA-based signature
Tiantan	Agilent 44 K array	212 glioma (AII 58, AIII 8, GBM 82, OII 18, OIII 10, OAII 21, OAIII 15)	Training set for searching for DRA-based signature
TCGA mRNA-seq	IlluminaHiseq_RNAseq	519 gliomas (AII 38, AIII 84, GBM 160, OII 83, OIII 54, OAII 55, OAIII 45)	Training set for searching for DRA-based signature
GSE4412	GPL96	85 gliomas(A 8,GBM 59,OA 7,O 11)	Validation set of DRA-based signature

The abbreviations for tumor types were derived from the source data: *A* astrocytoma, *AII* astrocytoma grade II, *AIII* astrocytoma grade III, *GBM* glioblastoma, *O* oligodendroglioma, *OII* oligodendroglioma grade II, *OIII* oligodendroglioma grade III, *OA* oligoastrocytoma, *OAII* oligoastrocytoma grade II, *OAIII* oligoastrocytoma grade III, *PA* pilocytic astrocytoma

### Differential co-expression analysis (DCEA) and differential regulation analysis (DRA)

We developed R package DCGL v2.0 for DCEA and DRA in our previous work [18, 19], which were used in the present study to detect differentially coexpressed genes and differentially regulated genes in glioma. We used R package limma for differential expression analysis [26].

### Clustering method

We applied nonnegative matrix factorization (NMF) clustering method [20] to get subgroups with distinct gene expression patterns. The number of clusters should keep all clusters as stable as possible, which can be checked by cophenetic correlation coefficient and heat map of clusters. Meanwhile, it should be as large as possible. (Additional file 1: Figure S1).

### Survival analysis

Patient’s overall survival time is calculated by counting the dates between surgery and death or the dates between surgery and last follow up. Kaplan-Meier survival curves were generated and analysed by using R package ‘survival’ [27]. *P* values were calculated by using the log-rank test to check the significant differences between the survival curves. Hazard ratio (HR) of one gene is often used to evaluate the potential risk of death related to high expression of this gene. If HR value of one gene is greater than 1, patient with high expression of this gene will have higher probability of having died. The calculation of genes’ hazard ratio was performed with ‘survcomp’ with survival time as the dependent variable [28, 29].

### Gene regulatory network modelling

The multivariant linear regression model proves to be able to infer gene regulatory relationships by gene expression profiles [30–32]. In our work, we constructed subtype-specific gene regulatory networks based on both forward predicted TF-target relationships and subtype-specific genes expression data by using the linear regression model. The true regulators of a particular gene and their regulation efficacies were determined by the stepwise linear regression.

## Results

### The identification of a three-TF glioma prognostic signature and its clinical relevance with the training set

In order to prioritize the regulators that are putatively causative to glioma, we first identified differentially regulated genes (DRGs) by using DCGL v2.0 [19] in GSE4290, and then chose the DRGs which were significant in both Targets’ Enrichment Density (TED) analysis and Targets’ DCL Density (TDD) analysis in DCGL v2.0 [19]. TED analysis evaluates enrichment of differential co-expression genes in a particular TF’s targets and TDD analysis measures density of differential co-expression links between a TF’s targets. TF might be more important or causative if it is significant or has higher ranking in both TED and TDD analysis. There are 87 significant TFs in TED analysis result and 79 significant TFs in TDD analysis result (Additional file 2: Table S1). We chose TFs that are significant in both these two analysis results. Therefore, six DRGs including AHR, NFIL3, ZNF423, MYC, MYCN and TAL1 were obtained. We listed their regulation targets based on TF2target library of DCGL v2.0 [19] which includes a set of candidate TF-target regulatory relationships. By assuming that these targets should be not only differentially expressed genes (DEGs) but also differentially co-expressed genes (DCGs), we acquired 253 links including 6 TFs and their 175 targets. However, some of these 175 genes are not differentially co-expressed with these 6 TFs. We then cut off these genes by using the differentially regulated links (DRL) analysis in DCGL v2.0. After sifting out DRLs, the links decreased to 93 with 6 TFs and their 82 targets. These 88 genes were considered as seed genes which are potentially related to glioma pathogenesis (Additional file 3: Table S2).

**Fig. 1:**
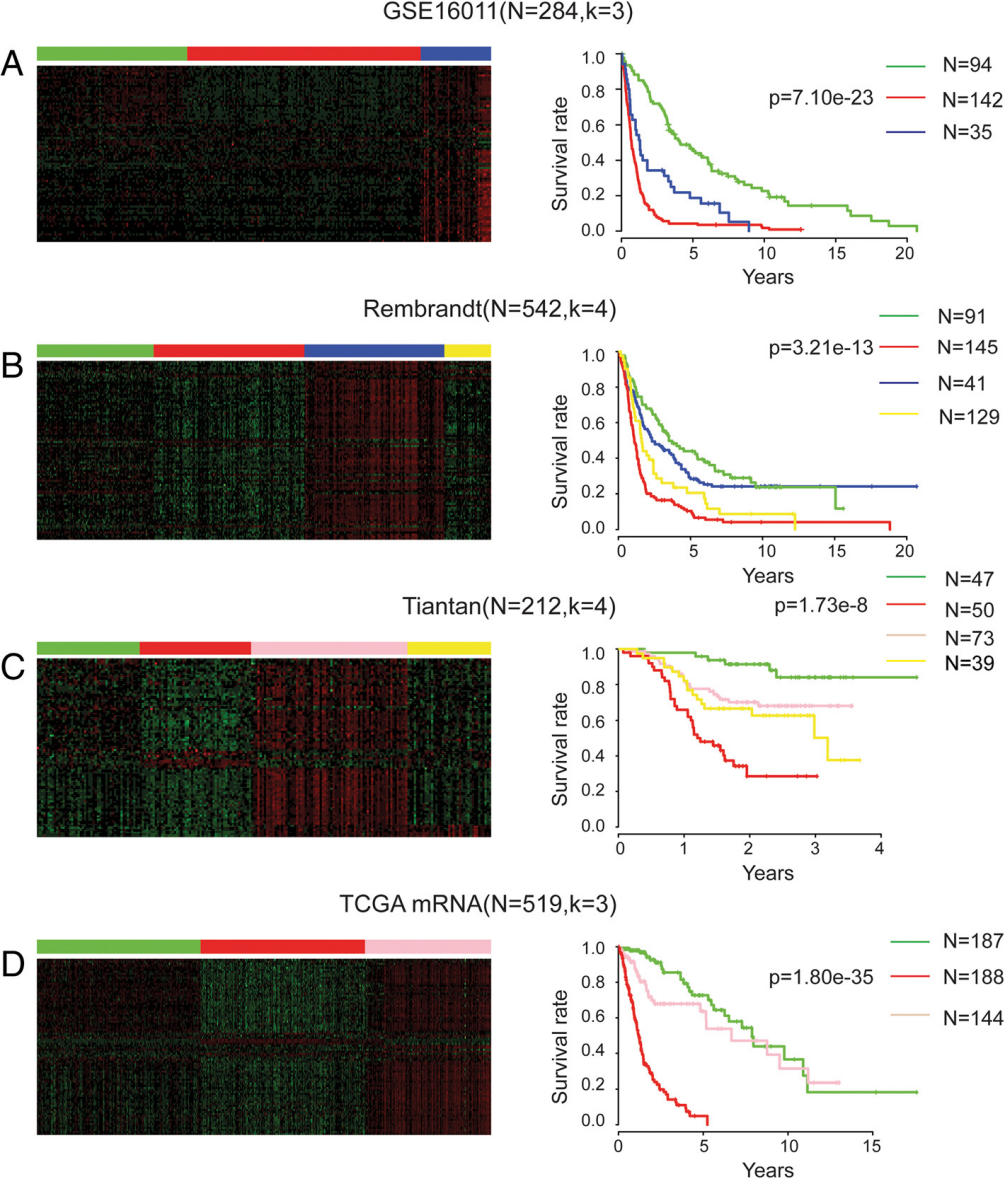
Clustering heat maps and survival analysis results with 88 seed genes in four data sets. The numbers of clusters (k) were determined by NMF based on the expression signatures of 88 potential glioma regulation related genes. Heat maps of 88 genes in glioma samples are shown on the left. **a**-**d** are respectively GSE16011, Rembrandt data set, Tiantan data set, and TCGA mRNA-seq data set. Kaplan-Meier survival curves of the overall survival for the patients from each molecular subtype are shown on the right. *P*-values of the survival curves were calculated by using log-rank tests. The same colour codes were used in the heat maps and the Kaplan-Meier survival curves in all datasets

Four glioma transcriptome data sets including GSE16011, Tiantan data set, Rembrandt data set and TCGA RNA-seq data set (the 2nd to 5thdata sets in Table 1), were used in our study as training sets to search for glioma classification signatures. Since a few of the 88 seed genes were not detected in some of the datasets, for example, LOC157627 was not in the Tiantan data set and six genes (C11orf9, EPB49, FRMPD4, LOC157627, NEFL, SLC7A14) were not in GSE16011, the expression values of the missing genes were taken as zero. By using NMF clustering method, these four data sets were divided into subgroups which have better prognosis, intermediate prognosis and worse prognosis associated with the clinical analysis (Fig. 1). When we looked into the best prognosis and worst prognosis clusters, we found that best prognosis clusters from the four data sets share 14 common genes which are ZNF423, ELAVL2, DOCK3, FGF13, GRM5, NRSN1, OPCML, PAK3, PDE2A, KCNQ5, RIMS2, RGS7, TAGLN3 and UNC5A; while worst prognosis clusters share 6 common genes including AHR, IGF2BP3, IGFBP2, IQGAP2, PLK2 and NFIL3. Notably, there are three transcription factors AHR, NFIL3 and ZNF423 in these 20 candidate genes.

**Fig. 2:**
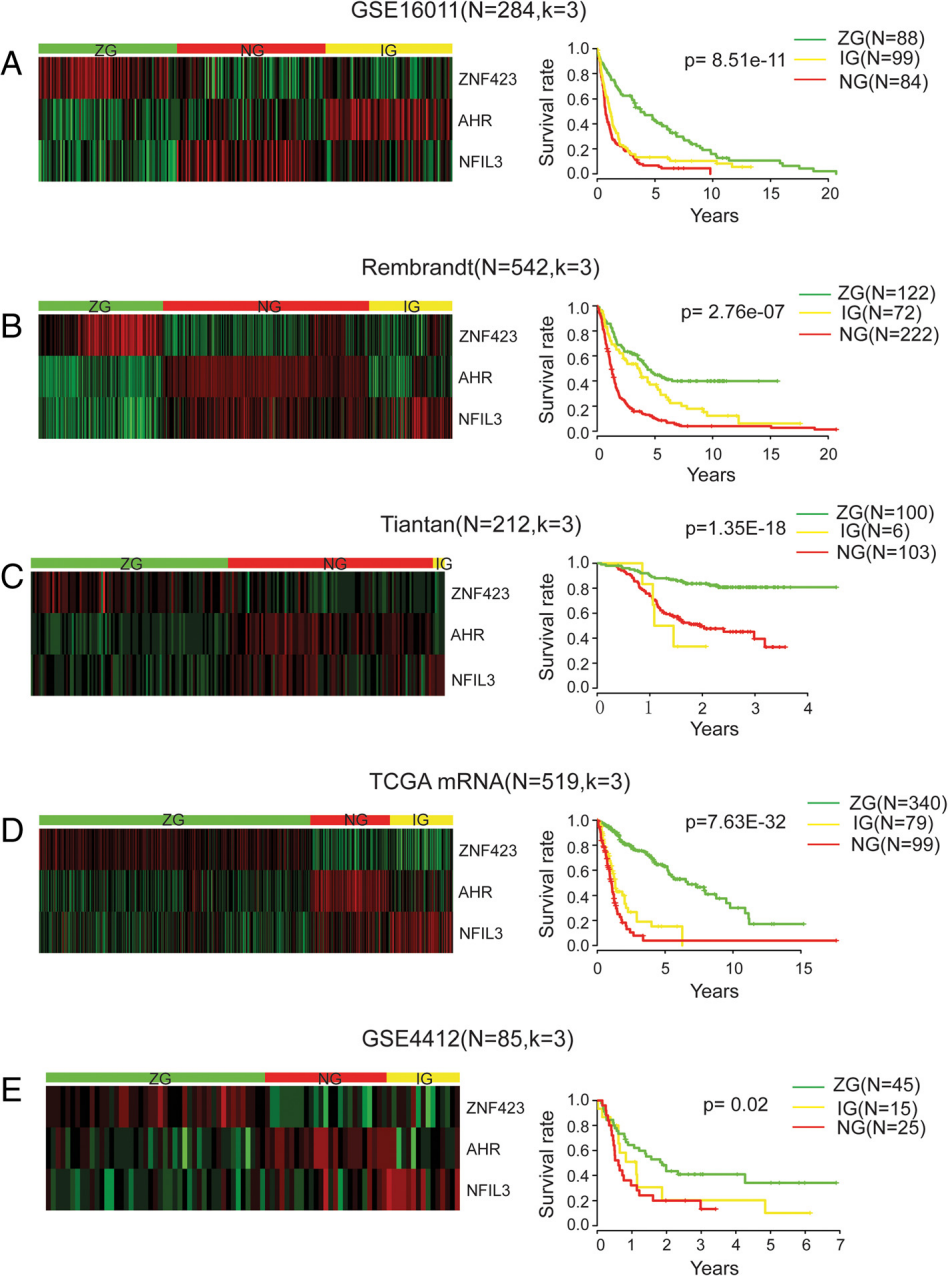
The clustering heat maps and survival analysis results with three-TF signature in five data sets. The numbers of clusters (k) were determined by NMF based on the expression signatures of 3 TFs. Heat maps of three-TF DRA signature in glioma samples are shown on the left. **a**-**e** are respectively GSE16011, Rembrandt, Tiantan, TCGA mRNA-seq and GSE4412 data sets. Kaplan-Meier survival curves of the overall survival for the patients from each molecular subtype are shown on the right. *P*-values of the survival curves were calculated by using log-rank tests. The same colour codes were used in the heat maps and the Kaplan-Meier survival curves of all datasets. The three colours green, red, yellow refer respectively to ZG, NG and IG subtypes

According to the basic understanding of gene regulation, changes of transcription factors are upstream events of cellular transcriptome. This is consistent with the observation that transcription factors are significantly enriched in reported glioma prognostic genes (Additional file 4: Table S3). And hazard ratios (Table 2 and Additional file 5: Table S4) are consistent with their reported functions in cancer progression. That is, hazard ratio of ZNF423 is less than 1 as high expression of ZNF423 is associated with better prognosis; hazard ratios of AHR and NFIL3 are greater than 1 as high expression of AHR and NFIL3 are associated with shorter survival time. Therefore, we checked if these three transcription factors (AHR, NFIL3 and ZNF423) could be a glioma prognosis-related signature by measuring its clinical relevance with NMF clustering method and survival analysis. Figure 2a-d show the results of the clustering heat map and survival analysis. The samples are divided into three subgroups for each data set according to the cophenetic correlation coefficient. The survival information between three subgroups are significantly different. For GSE16011 data set (Fig. 2a), the high expression value of ZNF423 is associated with the best prognosis group, and samples with high expression values of AHR and NFIL3 are divided into worst and intermediate prognosis groups. The survival curves of subgroups have significant differences (*p* = 8.51e-11) in this data set. In the Rembrandt data set which has the largest sample number, the best prognosis group has higher expression in ZNF423 and the clinical outcomes are differential as well (*p* = 2.76e-7) (Fig. 2b). The heat map of clustering result for Tiantan dataset shows similar gene expression patterns of ZNF423, AHR and NFIL3 (Fig. 2c). The statistic survival result shows three subgroups are different (*p* = 1.35e-18) despite that the survival curve of intermediate group is slightly mixed with the others according to its relatively small group number (6/212). TCGA RNA-seq data set has the same gene expression tendency as the above three data sets, and its subgroups are significantly different in survival information (*p* = 7.63e-32) (Fig. 2d). We named the subgroup with higher ZNF423 expression value as ZG group and that with higher expression values of NFIL3 and AHR as NG group. The intermediate group between ZG and NG groups is IG group. Ages at diagnosis in NG subtype are older than that in ZG subtype (Additional file 6: Table S5): 54.77 ± 13.5 in NG and 46.5 ± 13.4 in ZG of GSE16011 data set; 43.0 ± 12.7in NG and 39.0 ± 10.5 in ZG of Tiantan data set; 58.5 ± 13.5 in NG and 42.0 ± 14.3 in ZG of TCGA data set. Survival years of ZG groups are larger than that of NG groups: 3.5 ± 4.5 in ZG group and 0.7 ± 1.8 in NG group for GSE 16011 data set; 2.3 ± 0.9 in ZG group and 1.5 ± 0.8 in NG group for Tiantan data set; 3.5 ± 3.8 in ZG group and 1.2 ± 2.5 in NG group for Rembrandt data set; 1.0 ± 2.6 in ZG group and 0.7 ± 1.8 in NG group for TCGA data set.

**Table 2:** Hazard ratios of three TFs in four datasets (GSE16011, Rembrandt, Tiantan, TCGA mRNA datasets)

TFs	Gse16011		Rembrandt		Tiantan		TCGA mRNA		Reported functional remarks
	HR	*p*-value	HR	*p*-value	HR	*p*-value	HR	*p*-value	
ZNF423	0.188	4.81E-15	0.769	4.91E-09	0.4719	0.0014	0.627	5.46E-18	Overexpression of ZNF423 helps growth inhibition and differentiation. Neuroblastomas with low levels of ZNF423 show extremely poor outcome. [45, 46]
AHR	1.1818	0.001642	1.2669	6.45E-26	1.5729	1.68E-06	1.616	9.27E-16	AHR leads proliferation of Medulloblastoma cell. The pathway associated with AHR is active in human brain tumours with malignant progression and poor survival. [47–49]
NFIL3	1.394	1.20E-05	1.3529	5.31E-12	2.7649	2.18E-08	2.093	1.86E-15	NFIL3 is found to be overexpressed in different cancer types [50].

The statistics shows that DRA-signature subtypes overlap with morphologically defined glioma subtypes similarly in these data sets (Additional files 7, 8, 9, 10 and 11: Figure S2-S6). GBM samples are mainly clustered into NG and IG groups with worst prognosis: 81 (50.9 %) and 54 (34.0 %) over total 159 GBM samples are in NG and IG group in GSE16011 data set; 67 (81.7 %) and 4 (4.9 %) over total 82 GBM samples are in NG and IG group in Tiantan data set;178 (78.1 %) and 22 (9.6 %) over total 228 GBM samples are in NG and IG group in REM data set;77 (48.1 %) and 58 (36.3 %) over total 160 GBM samples are in NG and IG group in TCGA data set. Majority of other grade II and grade III glioma cases are divided to ZG group with best prognosis and the proportions for each data set are: 62 (56.9 %) out of 109 grade II and grade III samples are in ZG group in GSE16011 data set; 90 (69.2 %) out of 130 grade II and grade III samples are in ZG group in Tiantan data set; 315 (87.7 %) out of 359 grade II and grade III samples are in ZG group in TCGA data set. The distribution of samples crossing different subtypes indicates that molecular factors may vary within a certain traditional morphological subtype, which makes the diagnosis and treatment for molecular subtypes of glioma necessary replenishment for better prognosis of patients.

### Confirmation of the prognostic value of the three-TF signature in validation set

We validated the three-TF signature in the validation data set, GSE4412, which has 85 samples. By applying the NMF clustering method and survival analysis, we discovered three subgroups with significantly different patterns on both gene expression and prognosis result. Still, ZG group with higher gene expression value of ZNF423 has best prognosis while AHR and NFIL3 are highly expressed in NG and IG groups which have poorer prognosis (Fig. 2e). In spite of the smaller size of GSE4412 (*N* = 85) relative to four training data sets which have hundreds of sample, the survival curves of three subgroups are significantly different (*p* = 0.02). The statistic clinical information of subtypes of GSE4412 is listed in Additional file 11: Figure S6.

### Differential expression of the three-TF signature in glioma subtypes

**Fig. 3:**
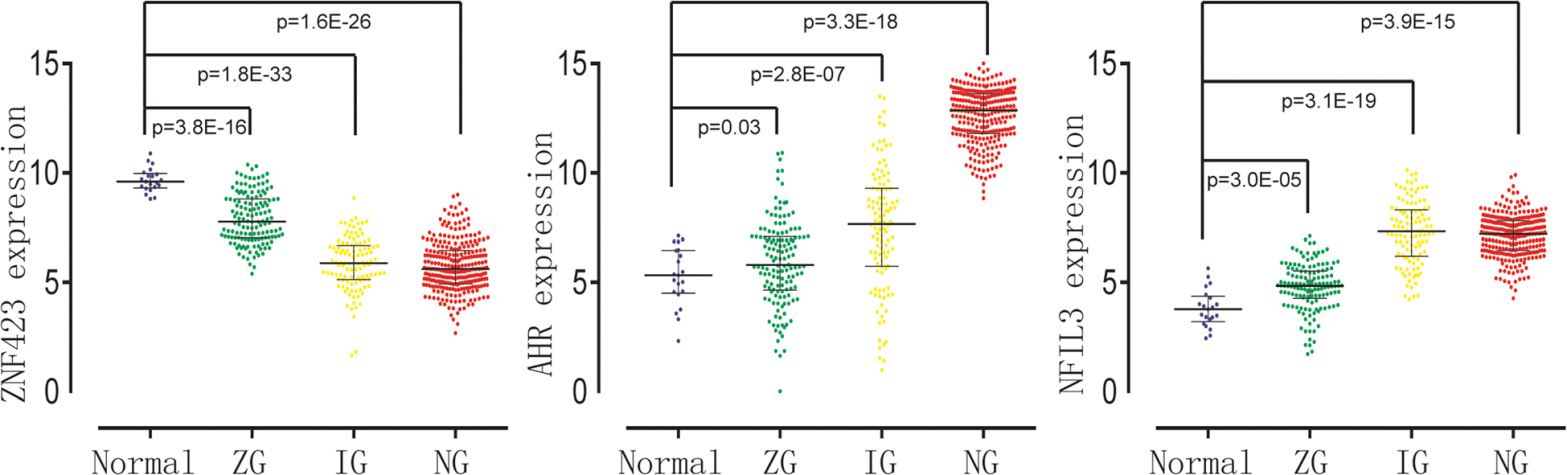
Expression values of ZNF423, AHR, NFIL3 in glioma subtypes in Rembrandt dataset. Each single point is the gene expression value of individual sample. Lines in the middle are the median expression values. The four colours blue, green, yellow and red represent respectively samples in normal, ZG, IG, NG subgroups

Since we obtained the glioma signature genes through differential coexpression analysis, which does not concern about the expression levels of individual genes, we checked the expression patterns of these three signature genes across the glioma subtypes. Rembrandt data set with normal sample data was used because it has the largest sample size (*N* = 542) and would reflect the glioma gene profile in a maximum effort. The expression values of AHR, NFIL3 and ZNF423 across glioma subtypes are significantly different (Fig. 3). These three genes are differentially expressed across subtypes in GSE16011 and the other three data sets as well (Additional file 12: Figure S7). This suggests that the differential co-expression analysis is a powerful complement to differential expression analysis in classification study. Vice versa, it is in essence the different expression value of signature genes that distinguish the glioma molecular subtypes.

### Comparison of the Three-TF signature and previously reported diffuse glioma biomarkers

Previously reported glioma biomarkers include 840-gene signature by TCGA [3], 1577-gene signature by CCGA [4], 69-EM/PM-gene signature by Sun et al. [6] and nine-gene signature by Bao et al. [5]. We still adopted GSE4412 as a validation data set. The 85 samples were clustered with these four signatures respectively by NMF method, and the survival curves were examined. The *p* value indicating the significant survival difference with four signatures in order are: 0.088, 0.023, 0.016, 9.24e-5 (Additional file 13: Table S6). The nine-gene signature has the best prognosis differentiation because clinical survival information was directly included in their regression model. This means our three-TF signature scheme which leads to significant *p* = 0.02 is comparable to the previous classification schemes, while our signature is with the least number of genes, offering more feasibility for clinical application. Additionally, all of three signature genes are transcription factors, which might help to explain the regulation mechanisms underlying glioma carcinogenesis. It is noticeable that our signature genes do not overlap with any of the four previously reported signatures.

In order to compare the drug target relevance of our DRA-based signature and previously reported signatures, we evaluated the enrichment of the signatures in the 1318 FDA-proved-drug target genes in Open Data Drug & Drug Target Database (DrugBank) [33]. In GSE4290 data set, there are 1277 (6.3 %) drug target genes out of total 20,284 expressed genes, while our signature has 1 drug target AHR over total 3 genes (enrichment significant *p* = 0.028) (Additional files 13 and 14: Table S6 and S7). The CCGA 1577-gene signature has 168 drug targets (*p* = 0). The TCGA 840-gene signature has 91 drug targets (*p* = 4.1e-10). The 69-EM/PM-gene signature has 3 drug targets which are PDGFRA, MMP16 and EGFR (*p* = 0.25). There is no drug target gene in nine-gene signature. The significant enrichment of our three-TF signature in drug target genes indicates its superior potential in medical treatment although some reported signatures with thousands of genes have better drug target enrichments.

### Exploratory analysis on relevance of the DRA-based three-TF signature to glioma pathogenesis

**Fig. 4:**
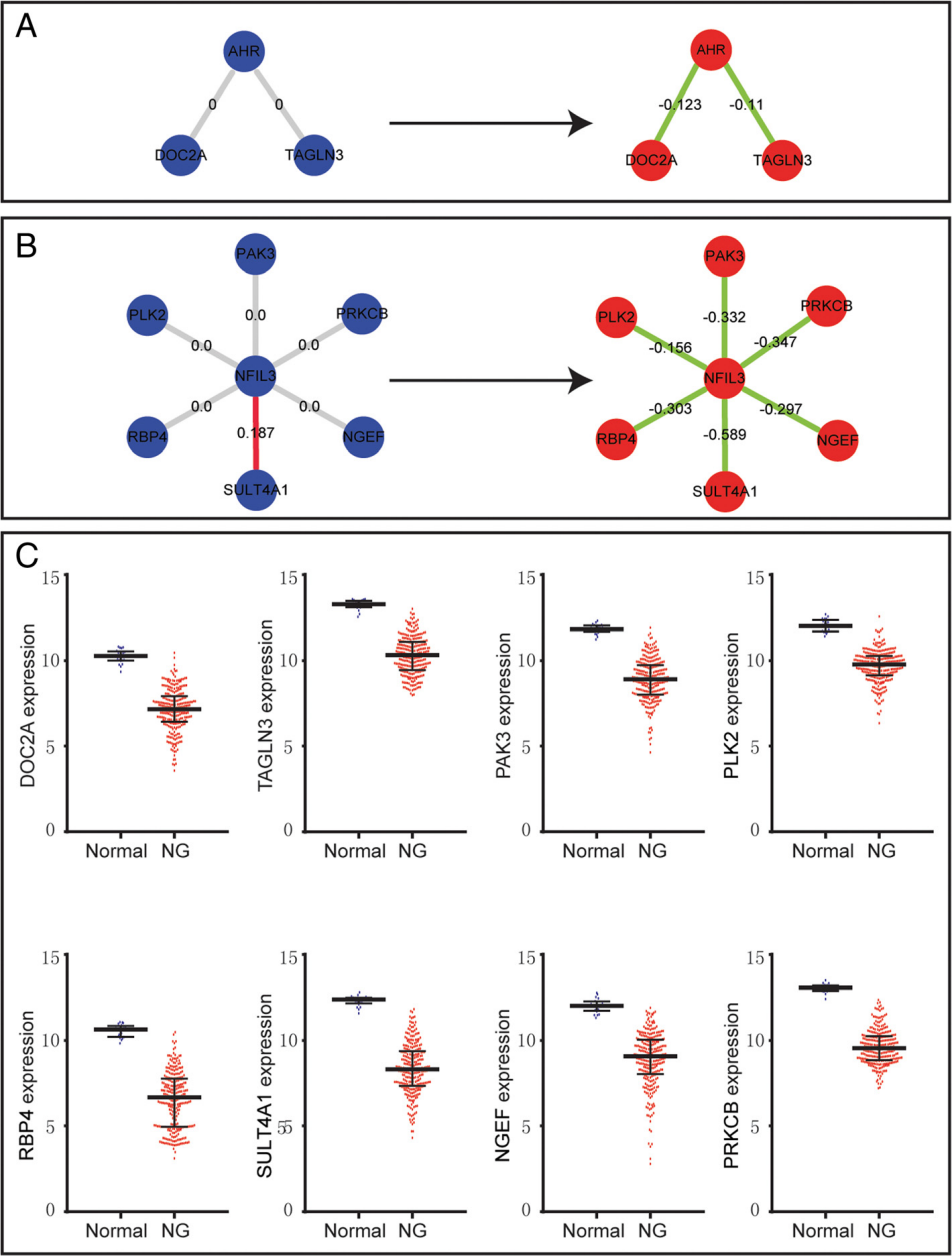
DRA-signature genes and their notable regulatory networks in different glioma molecular subtypes. **a**-**b** Differential regulations of AHR and NFIL3: the regulatory relationships of normal condition (*blue*) were shown in left, the regulations of NG group (*red*) were shown in right. The labels of edge were indicators to measure the relationship between TFs and targets. Red and green represent positive regulatory efficiency and negative regulatory, grey represent no relationship between TFs and targets. Higher absolute label value means stronger regulations. **c** The expression values of the target genes shown in (**a**) and (**b**) in normal condition (*blue*) and NG group (*red*), each single point is the gene expression value of individual sample. Lines in the middle were the median expression values

We investigated subtype-specific gene regulatory relationships in Rembrandt data set by using regulatory network modelling method [30–32]. According to the differential networking information and the previously reported signalling knowledge, we explored the relevance of our DRA-based three-TF signature to glioma pathogenesis and highlighted some significant regulatory relationships in glioma subtypes (Fig. 4). And the global significant regulatory relationship of 88 seed genes was shown in Additional file 15: Figure S8.

As mentioned above, highly expressed AHR is associated with the worse-prognosis group. We checked the difference of the regulation relationships of AHR and its targets among 88 seed genes (Additional file 3: Table S2) between NG group and normal sample. In Fig. 4a, it was found that AHR does not regulate DOC2A in normal samples while negatively regulates DOC2A in NG group, which was confirmed by the lower expression level of DOC2A in NG group. Since DOC2A has been reported as a suppressor gene in many carcinomas such as colorectal cancer and urothelial cancer [34, 35], we suggest AHR might promote glioma progression through inhibiting the tumor suppressor gene, DOC2A. Another regulatory relationship AHR-TAGLN3 is notable as well. Similarly, AHR negatively regulates TAGLN3 in NG group (Fig. 4a) and TAGLN3 has lower expression value in NG group than that in normal samples. TAGLN2, which is homologous gene of TAGLN3, was reported to have tumour-suppressive function in bladder cancer [36]. TAGLN3 might be another important role in promoting glioma progression which leads to worse prognosis.

We also noticed that NFIL3 with high expression is featured in the worse-prognosis subtype and it regulates many cancer related genes (Fig. 4b). For example, PLK2, a tumour suppressor gene [37], is negatively regulated by NFIL3 and has lower gene expression value in NG group. RBP4, a tumor suppressor in ovarian cancer [38], is negatively regulated by NFIL3 and has lower gene expression value in NG group. Similarly, SULT4A1, with lower expression in children brain tumor [39], is regulated positively by NFIL3 in normal samples while negatively by NFIL3 in NG group. The negative regulation relationship between NFIL3 and NGEF in NG group, and the lower expression of NGEF was also observed, which is consistent with the previous report [40].

Interestingly, another two regulation targets of NFIL3, PRKCB and PAK3, were reported as oncogenes [41, 42], however, they are negatively regulated by NFIL3 and show lower expression levels in NG group. More detailed function studies on these two oncogenes seem to be required to elucidate their functions in glioma carcinogenesis.

## Discussion

Using integrated gene co-expression analysis approach, we identified three prognostic biomarkers to cluster the glioma into three molecular subtypes (ZG, NG and IG subtypes) with significantly different clinical outcomes, gene expression patterns and transcriptional regulation patterns. Among the subtypes, ZG is featured with higher expression of ZNF423 and has better prognosis with younger age at diagnosis; NG is associated with higher expression of NFIL3 and AHR, and has worse prognosis with elder age at diagnosis. This three-TF signature was validated by independent glioma data set for its prognostic value. More and more biomarkers of cancers including glioma are discovered with rapid development of high-throughput technologies and large-scale genetic data generated increasingly. These multidimensional biomarkers such as mutations and gene expression patterns supplement histology-dominated profiles of glioma and other cancers [43]. In the meantime, they have been driving the development of precision medicine in cancer, which aims to analyse individual patient’s disease at molecular level and conduct more targeted treatments.

However, large amount of methods searching for biomarkers highly rely on prior knowledge such as directly testing whether there is an association between the survival results and the well-known tumor related genes, inefficiently making use of the high-throughput data that contains valuable correlation information. Moreover, most of biomarkers are identified solely according to their statistical associations with clinical outcomes, therefore they have limited ability to pursue underlying pathogenic mechanisms.

In current efforts to carcinogenesis studies, lots of attention has been paid on differential gene expression. The functional-relevant, differentially expressed genes (DEG) are always selected to explain regulation mechanisms underlying pathogenesis. However, it has been well accepted that cancer originates from genomic changes in genes regulating cell growth and differentiation, which induces abnormal expression of a large number of genes, and over-activation of cell proliferation [44]. That is, DEGs are the consequences of differential regulation mechanisms instead of the causes of phenotypic changes. Accordingly, DEG-based signatures are more likely to be ‘associated’ with a certain phenotype, but less relevant to the causal mechanisms. In our previous work, we developed a series of differential co-expression analysis (DCEA) and differential regulation analysis (DRA) methods, which aim to explore gene regulation changes, or differential gene regulation [17–19]. DCEA has been considered more promising in identifying differential regulation mechanisms of phenotypic changes than differential expression analysis (DEA) [16]. In our present work, we combined DRA, DCEA and DEA to select seed genes for signature identification, instead of only investigating differentially expressed genes as previous studies did [12–15]. Benefiting from the use of DRA and DCEA, the searching space of signature genes was narrowed down to the genes most relevant to differential regulation. Our DRA-based three-TF signature genes have been proved to be more causal and help to generate testable hypothesis on glioma carcinogenesis. Contrarily, in the method which defines nine-gene signature based on association between differential expression and clinical information [5], although its *p*-value of survival curves is the smallest and the number of its signature genes is quite small, the nine genes deliver limited information on glioma carcinogenesis.

It is noticeable that choosing the prognostic biomarkers as less as possible is able to make clinical research more effective. In our present work, we chose only transcription factors from the 20 candidate genes as glioma signature, since TFs are believed to be up-stream factors in carcinogenesis and have potential to be effective drug targets in treatments. To evaluate the validity of this procedure, we randomly generated a new three-gene-signature from 20 genes listed in Additional file 4: Table S3 and conduct the survival analysis of their molecular subtypes (Additional file 16: Table S8). It was found that *p*-value of our three-TF signature is significant and smaller than the average of *p*-values of randomly generated ones (Additional file 17: Figure S9). Additionally, the hazard ratio values of the three TFs (Table 2) match with previous knowledge about their functions. All above means these three TFs have sufficient power to classify prognostic molecular subtypes, indicating that transcription factors are priorities in biomarker candidates. Based on our inferred differential networking information and the previously reported signalling knowledge around our signature genes, we generated testable hypotheses on the roles of AHR and NFIL3 in glioma carcinogenesis. We also proposed some testable hypotheses on the roles of AHR and NFIL3 in glioma carcinogenesis which are worthy of further experimental investigations.

## Conclusions

In conclusion, our classification scheme, which is based on differential co-expression and differential regulation analysis, is able to predict the prognosis of glioma by only three genes. Our research explores the glioma molecular mechanism at transcriptional regulation level and provides potential drug targets for different glioma molecular subtypes. This integrated approach is extendable to other cancer researches for the identification of complex disease biomarkers with hints for not only diagnosis, but also pathogenesis.

## Abbreviations

DCEA, differential coexpression analysis; DEA, differential expression analysis; DEG, differentially expressed genes; DRA, differential regulation analysis; HR, hazard ratio; NMF, nonnegative matrix factorization; TF, transcription factor

## Additional files

Additional file 1: Figure S1. Cophenetic correlation coefficients of nonnegative matrix factorization (NMF) clustering method with 88 seed signatures. (TIF 6064 kb) Additional file 2: Table S1. Significant TFs of TDD and TED in GSE4290. (XLSX 15 kb) Additional file 3: Table S2. Information of 88 seed genes. (XLSX 13 kb) Additional file 4: Table S3. TF enrichment in reported prognostic featured genes. (XLSX 12 kb) Additional file 5: Table S4. Hazard ratio values of 17 non-TF in 20 target genes. (XLSX 14 kb) Additional file 6: Table S5. Tukey’s test for ages between molecular subtypes in four data sets. (XLSX 11 kb) Additional file 7: Figure S2. Comparison of 3-TF signature subtypes and morphologically defined glioma subtypes in GSE16011 data set. (TIF 2970 kb) Additional file 8: Figure S3. Comparison of 3-TF signature subtypes and morphologically defined glioma subtypes in Rembrandt data set. (TIF 2182 kb) Additional file 9: Figure S4. Comparison of 3-TF signature subtypes and morphologically defined glioma subtypes in Tiantan data. (TIF 2631 kb) Additional file 10: Figure S5. Comparison of 3-TF signature subtypes and morphologically defined glioma subtypes in TCGA mRNA data set. (TIF 2772 kb) Additional file 11: Figure S6. Comparison of 3-TF signature subtypes and morphologically defined glioma subtypes in GSE4412 data set. (TIF 2051 kb) Additional file 12: Figure S7. The differential expression patterns of three TF in glioma subtypes in four data sets. (TIF 1005 kb) Additional file 13: Table S6. Comparison of 3-TF signature and reported gene expression signatures. (XLSX 8.9 kb) Additional file 14: Table S7. Drug targets in 3-TF signature and reported gene expression signatures. (XLSX 13 kb) Additional file 15: Figure S8. Regulatory networks of three TFs in subtypes of Rembrandt data set. (TIF 2278 kb) Additional file 16: Table S8. Survival analysis of clustering results by three-random-gene signatures. (XLSX 80.4 kb) Additional file 17: Figure S9. The *p*-value distribution of survival analysis of clustering results by three-random-gene signatures. (TIF 995 kb)
