# Supplementary figures and images for: A novel integrated gene coexpression analysis approach reveals a prognostic three-transcription-factor signature for glioma molecular subtypes
Source: BMC Syst Biol. 2016 Aug 26;10(Suppl 3):71. doi: 10.1186/s12918-016-0315-y (PMC5009532; doi:10.1186/s12918-016-0315-y)

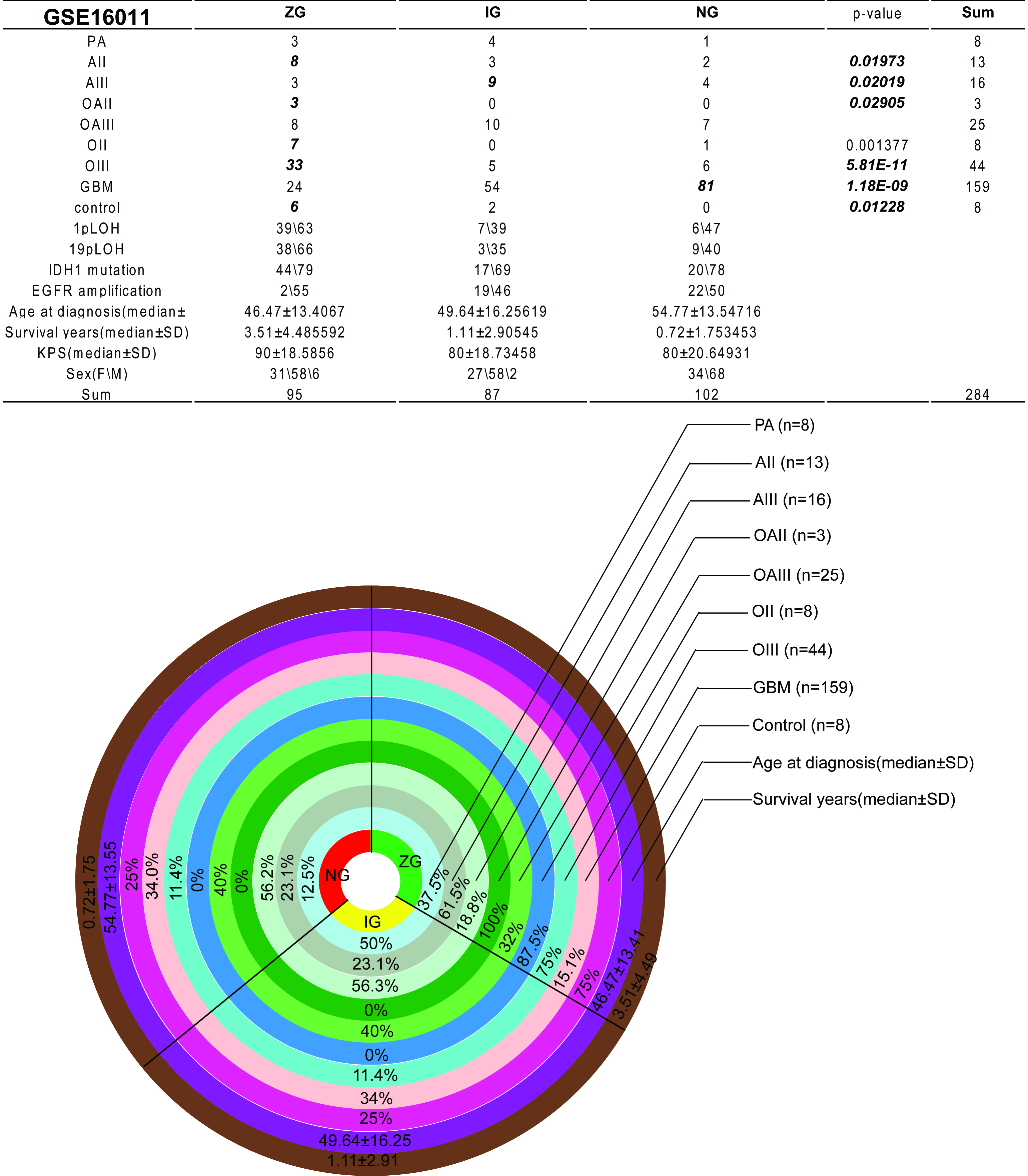

Supplement: Additional file 7: Figure S2. — Comparison of 3-TF signature subtypes and morphologically defined glioma subtypes in GSE16011 data set. (TIF 2970 kb) [file 12918_2016_315_MOESM7_ESM.tif]

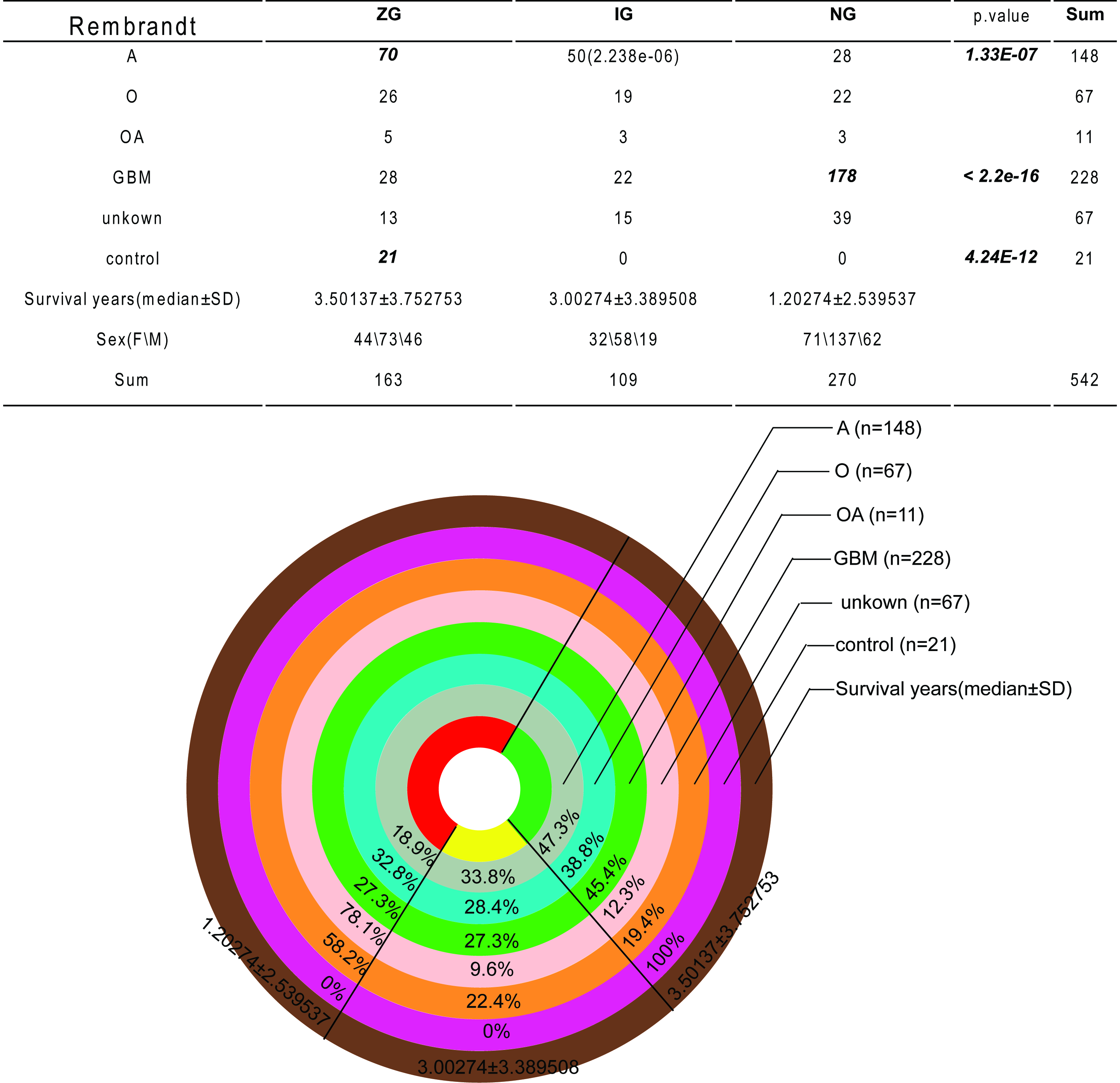

Supplement: Additional file 8: Figure S3. — Comparison of 3-TF signature subtypes and morphologically defined glioma subtypes in Rembrandt data set. (TIF 2182 kb) [file 12918_2016_315_MOESM8_ESM.tif]

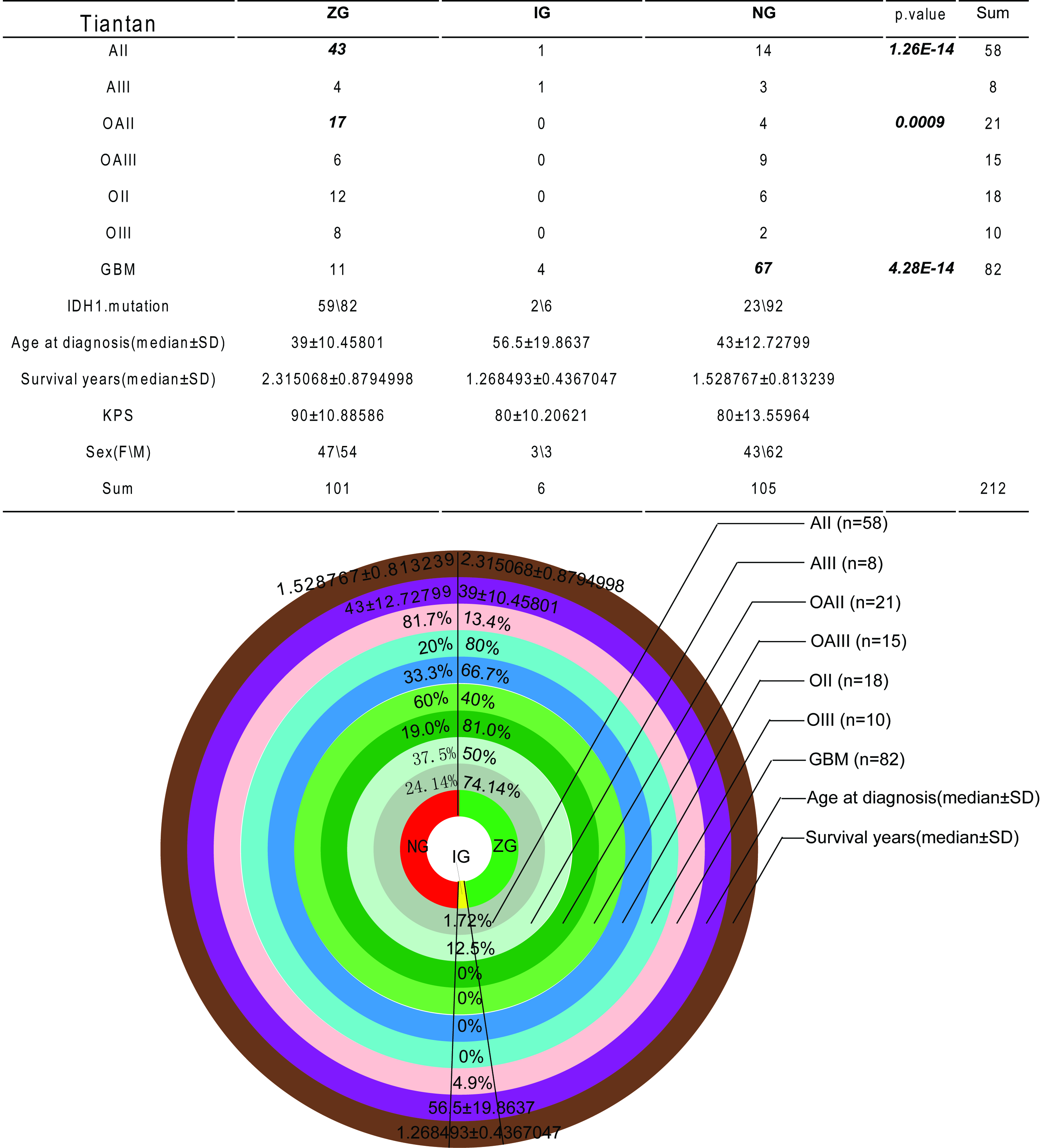

Supplement: Additional file 9: Figure S4. — Comparison of 3-TF signature subtypes and morphologically defined glioma subtypes in Tiantan data. (TIF 2631 kb) [file 12918_2016_315_MOESM9_ESM.tif]

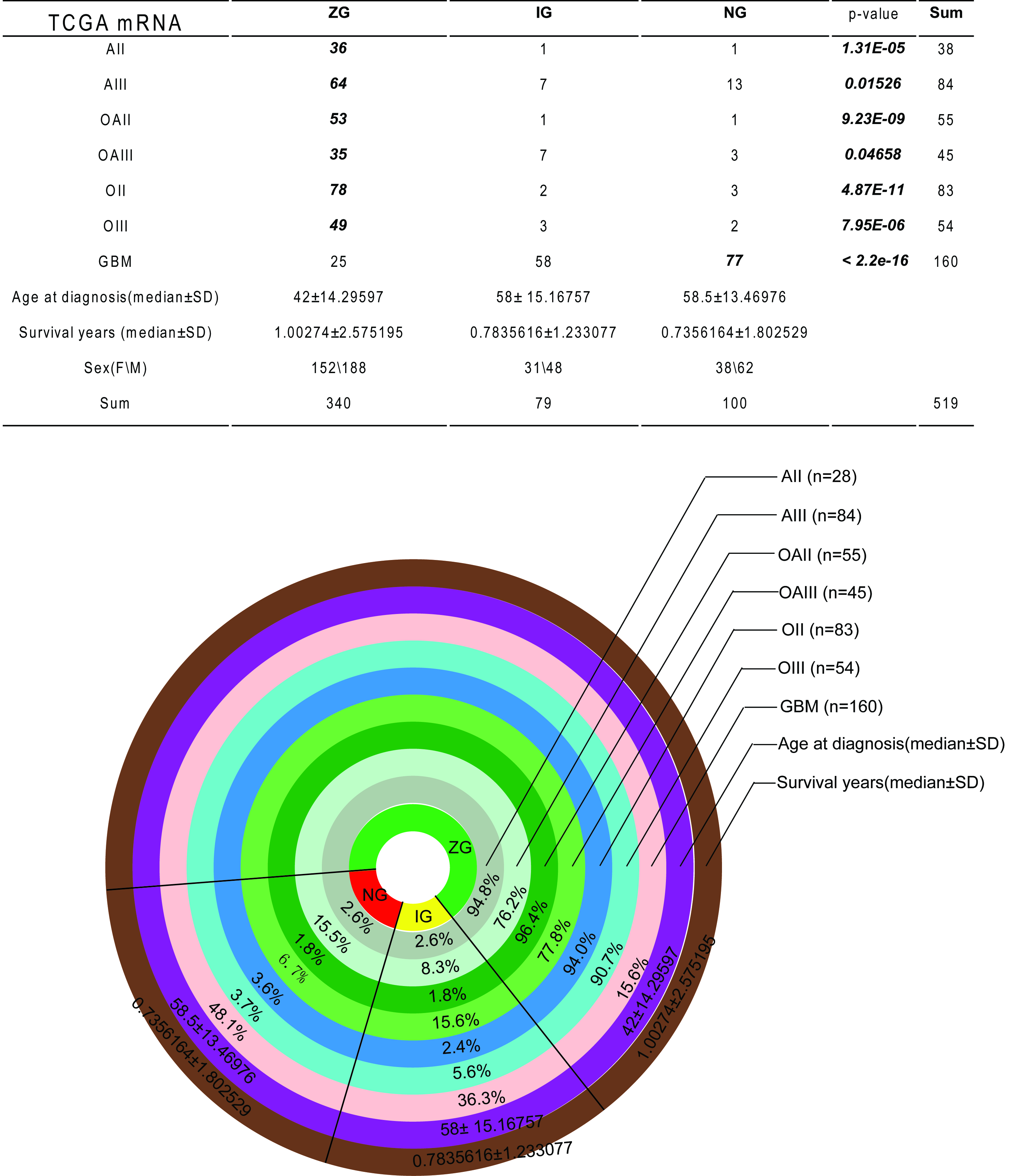

Supplement: Additional file 10: Figure S5. — Comparison of 3-TF signature subtypes and morphologically defined glioma subtypes in TCGA mRNA data set. (TIF 2772 kb) [file 12918_2016_315_MOESM10_ESM.tif]

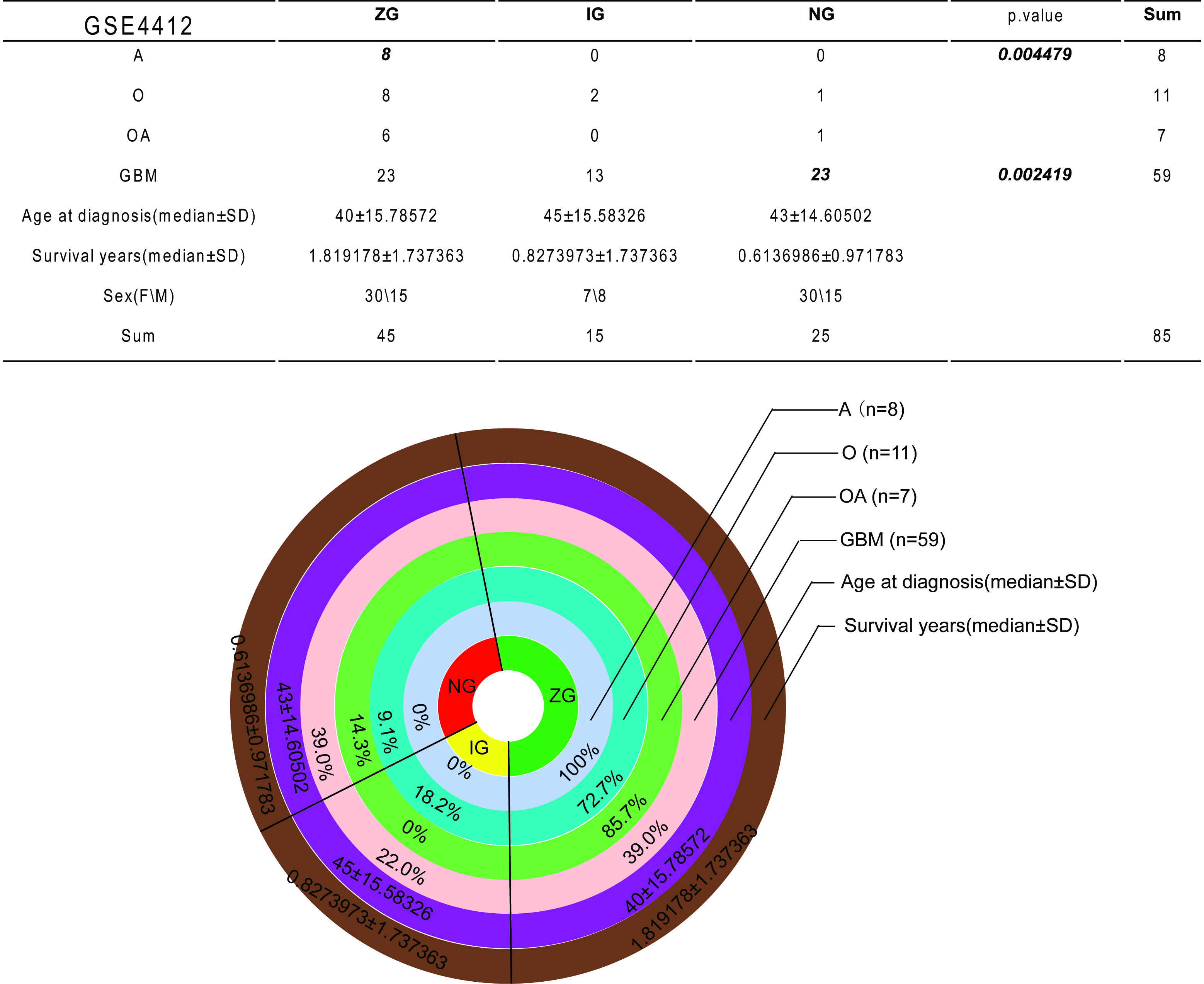

Supplement: Additional file 11: Figure S6. — Comparison of 3-TF signature subtypes and morphologically defined glioma subtypes in GSE4412 data set. (TIF 2051 kb) [file 12918_2016_315_MOESM11_ESM.tif]

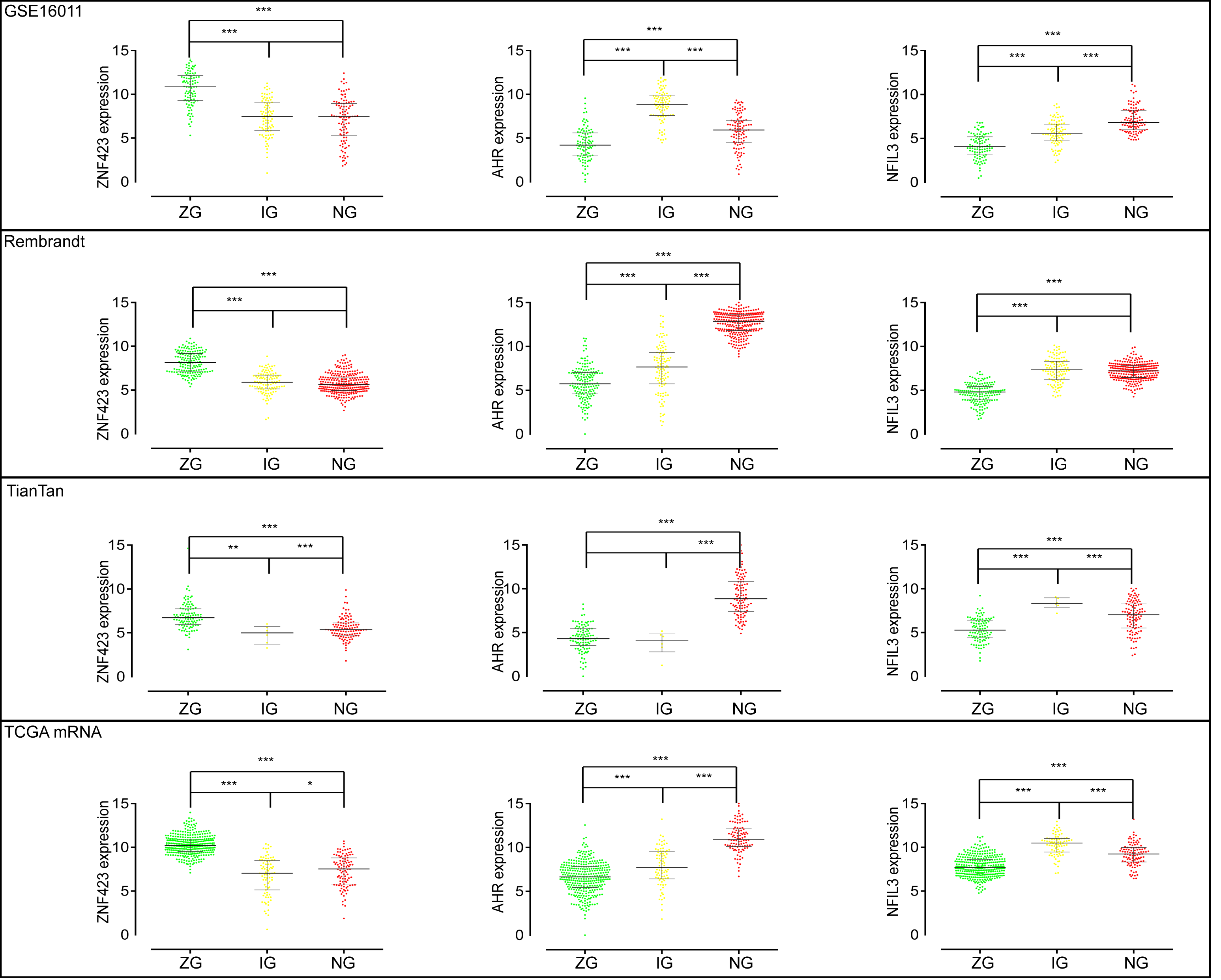

Supplement: Additional file 12: Figure S7. — The differential expression patterns of three TF in glioma subtypes in four data sets. (TIF 1005 kb) [file 12918_2016_315_MOESM12_ESM.tif]

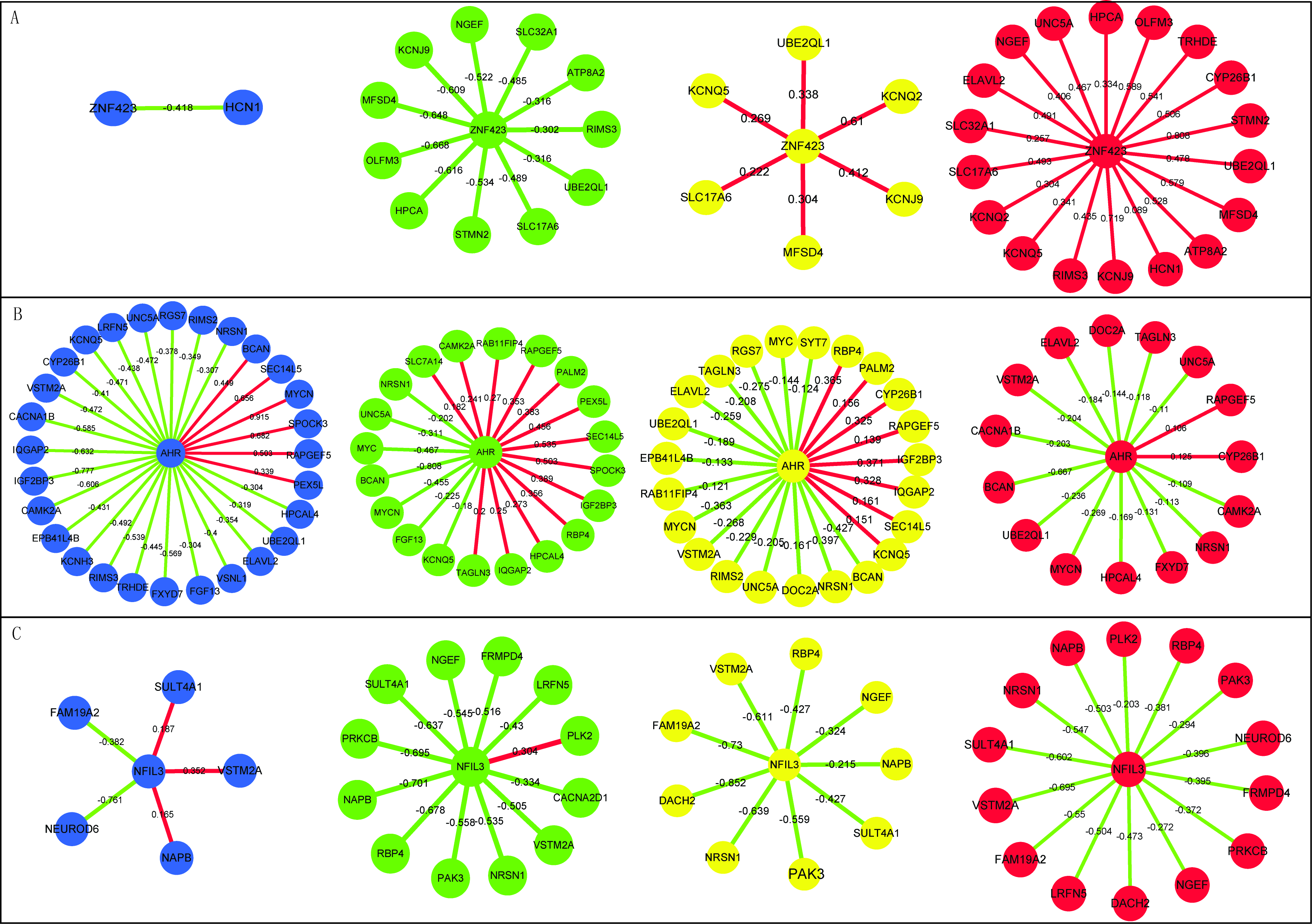

Supplement: Additional file 15: Figure S8. — Regulatory networks of three TFs in subtypes of Rembrandt data set. (TIF 2278 kb) [file 12918_2016_315_MOESM15_ESM.tif]

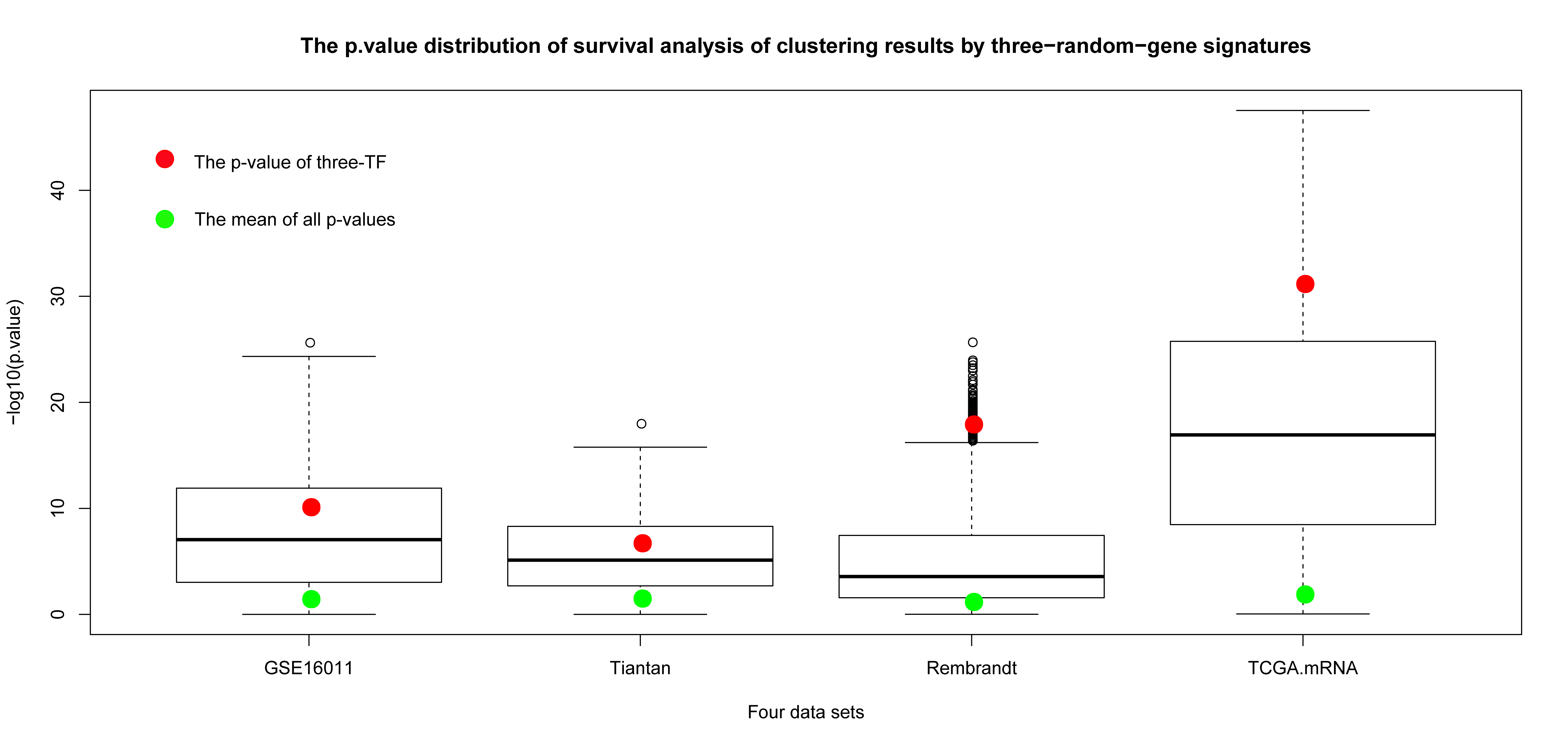

Supplement: Additional file 17: Figure S9. — The p-value distribution of survival analysis of clustering results by three-random-gene signatures. (TIF 995 kb) [file 12918_2016_315_MOESM17_ESM.tif]
